# Intrathoracic Gastric Perforation Secondary to Strangulated Recurrent Hiatal Hernia: A Case Report of Diagnostic and Therapeutic Challenges

**DOI:** 10.7759/cureus.85750

**Published:** 2025-06-11

**Authors:** Larissa Silva Coimbra, Matheus de Oliveira Santos, Giovanna Lima Labate, Aline Sardow Pereira, Thiago Henrique Sigoli Pereira

**Affiliations:** 1 Division of Trauma and Acute Care Surgery, Hospital das Clínicas, Ribeirão Preto Medical School, University of São Paulo (FMRP-USP), Ribeirão Preto, BRA

**Keywords:** damage control surgery, gastric perforation, hiatal hernia, strangulation, thoracic drainage

## Abstract

This report discusses a rare and challenging case of intrathoracic gastric perforation secondary to a recurrent strangulated hiatal hernia. The patient, a 52-year-old female with a prior history of thoracic and laparoscopic surgery for hiatal and diaphragmatic hernia, presented with epigastric and chest pain, which progressively led to hemodynamic shock. Initially, a pulmonary infection with parapneumonic pleural effusion was suspected. However, further imaging, including a contrast-enhanced computed tomography (CT) scan, revealed bilateral pleural effusion and herniation of the stomach with perforation into the thoracic cavity, which required emergency surgical intervention. Subtotal gastrectomy was performed, followed by peritoneostomy. Postoperatively, the patient was admitted to the intensive care unit. A few days later, she underwent peritoneostomy revision and Roux-en-Y gastrojejunostomy reconstruction. After clinical improvement, she was transferred to the general ward and subsequently discharged, with outpatient follow-up.

## Introduction

Hiatal hernia is defined as the herniation of intra-abdominal structures, most commonly the stomach, through the esophageal hiatus of the diaphragm into the mediastinum [1]. It is a relatively common condition, particularly among individuals over 50 years old, with an estimated prevalence of up to 60% in this age group, although many cases remain asymptomatic and undiagnosed [2]. Hiatal hernias are typically classified into four types: Type I (sliding hernia) accounts for the majority of cases and involves the gastroesophageal junction migrating above the diaphragm; Types II-IV are considered paraesophageal hernias, with varying degrees of gastric and other abdominal organ displacement, and carry a higher risk of complications such as incarceration, strangulation, and perforation [3,4].

Complicated hiatal hernias, although rare, present significant clinical challenges. Strangulation occurs when the herniated gastric wall undergoes vascular compromise due to constriction at the hiatus, leading to ischemia and potential perforation. This sequence can precipitate life-threatening conditions, including tension hydropneumothorax, mediastinitis, and severe sepsis [5,6]. In such cases, the nonspecific nature of initial clinical manifestations - often mimicking pulmonary infections, cardiac events, or pleural diseases - may delay diagnosis, thereby worsening prognosis. Imaging studies, particularly computed tomography (CT) of the chest and abdomen with oral contrast, are essential tools for early detection, especially in the context of pleural effusion with suspected gastrointestinal origin [7,8].

Management of complicated hiatal hernias with gastric perforation is emergent and requires prompt surgical intervention. The surgical strategy should be tailored according to the patient's hemodynamic stability, the extent of contamination, and the viability of the herniated organs [9]. When patients present in critical condition, a damage control approach - prioritizing rapid control of contamination and stabilization over definitive anatomical repair - is indicated [10]. 

This case report describes a rare and severe presentation of intrathoracic gastric perforation secondary to recurrent strangulated hiatal hernia, initially misdiagnosed as a pulmonary infection, highlighting the diagnostic and therapeutic challenges associated with this entity. 

## Case presentation

**Figure 1:**
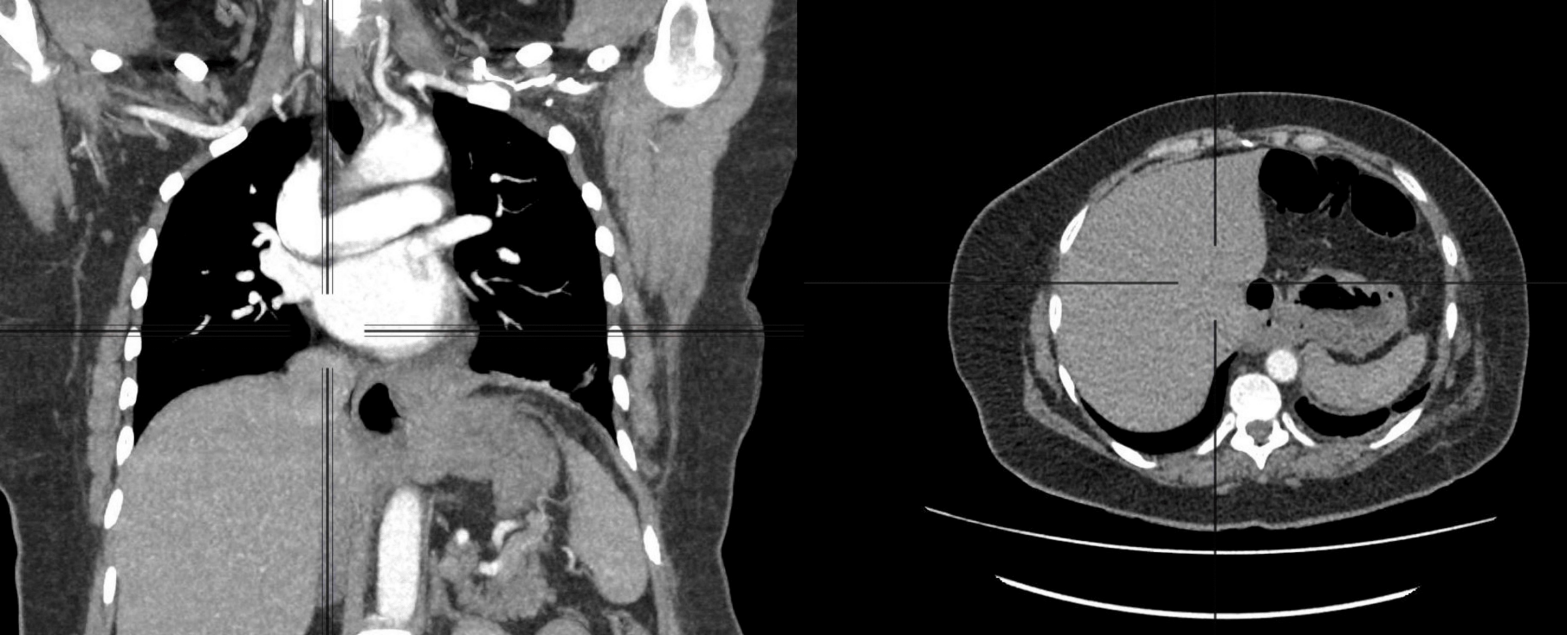
Control computed tomography, performed four months postoperatively, shows no evidence of recurrence or other abnormalities

A 52-year-old female patient with a history of hiatal hernia repair performed via videolaparoscopy seven months earlier was discharged from outpatient follow-up after a control CT, performed four months postoperatively, showed no evidence of recurrence or other abnormalities (Figure 1). The previous repair consisted of reduction of the herniated stomach, cruroplasty, and construction of a partial posterior fundoplication, with fixation of the valve to the right and left diaphragmatic crura.

Three months after the control CT, the patient was admitted to a low-complexity regional hospital with complaints of epigastric pain and constrictive chest pain. She progressed with ventilatory discomfort and hemodynamic shock. The initial working diagnosis was sepsis of pulmonary origin, due to suspected pneumonia with associated parapneumonic pleural effusion.

**Figure 2:**
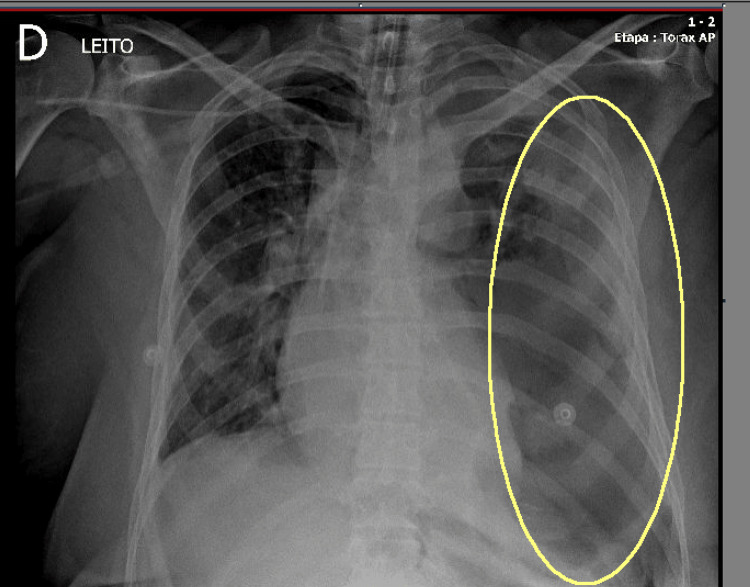
Bedside chest X-ray showing pleural effusion (transparent yellow oval)

She was transferred to a high-complexity hospital one day after admission to the previous hospital. Upon arrival, she was under orotracheal intubation, on mechanical ventilation in assist-control mode, sedated, and receiving norepinephrine (0.5 mcg/kg/min) in association with vasopressin. A sepsis protocol was initiated, including broad-spectrum antibiotic therapy and neuromuscular blockade due to ventilatory asynchrony. Laboratory tests revealed acute kidney injury, consistent with KDIGO (Kidney Disease Improving Global Outcomes) Stage 3; elevated C-reactive protein; and blood gas analysis showed combined metabolic and respiratory acidosis with elevated lactate levels. A bedside chest X-ray demonstrated pleural effusion (Figure 2).

**Figure 3:**
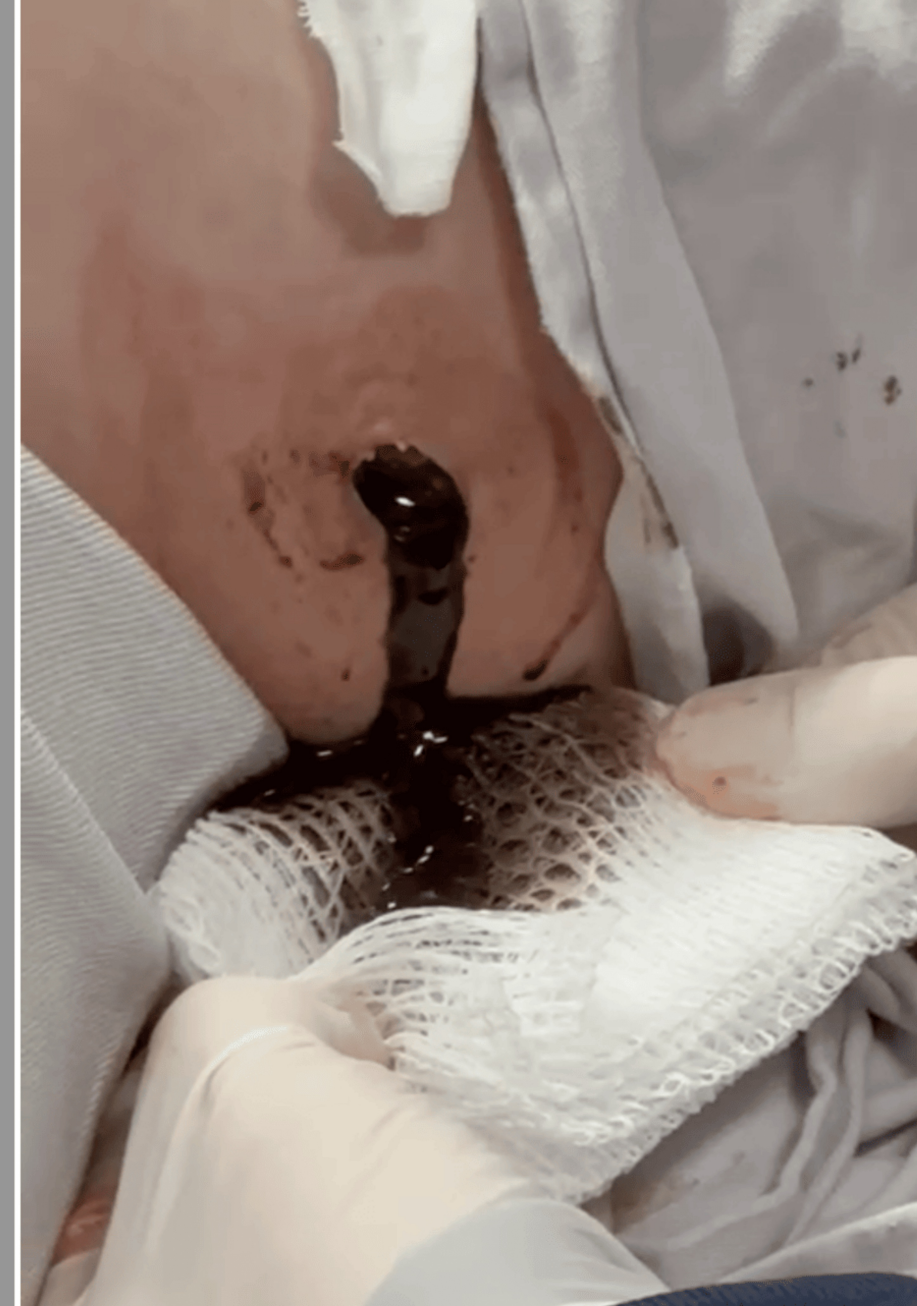
Appearance of left thoracic drainage showing presence of enteric content

**Figure 4:**
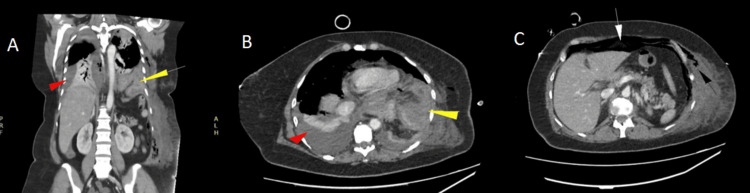
Contrast-enhanced CT images A) Coronal slice showing herniated stomach (yellow arrow) and pleural effusion (red arrow); B) Axial slice showing herniated stomach (yellow arrow) and pleural effusion (red arrow); C) Axial slice showing pneumoperitoneum (white arrow) and subcutaneous emphysema (black arrow). CT, computed tomography

The team opted for intercostal tube drainage, performed in the fifth intercostal space at the anterior axillary line, with immediate output of 1,200 mL of enteric content (Figure 3). After drainage, the patient showed improvement in ventilatory mechanics and blood pressure control, although high doses of vasopressors were still required. Following initial stabilization, a contrast-enhanced CT revealed bilateral pleural effusion, predominantly on the left side (Figure 4), as well as herniation of the stomach, thoracic subcutaneous emphysema, and large-volume pneumoperitoneum.

**Figure 5:**
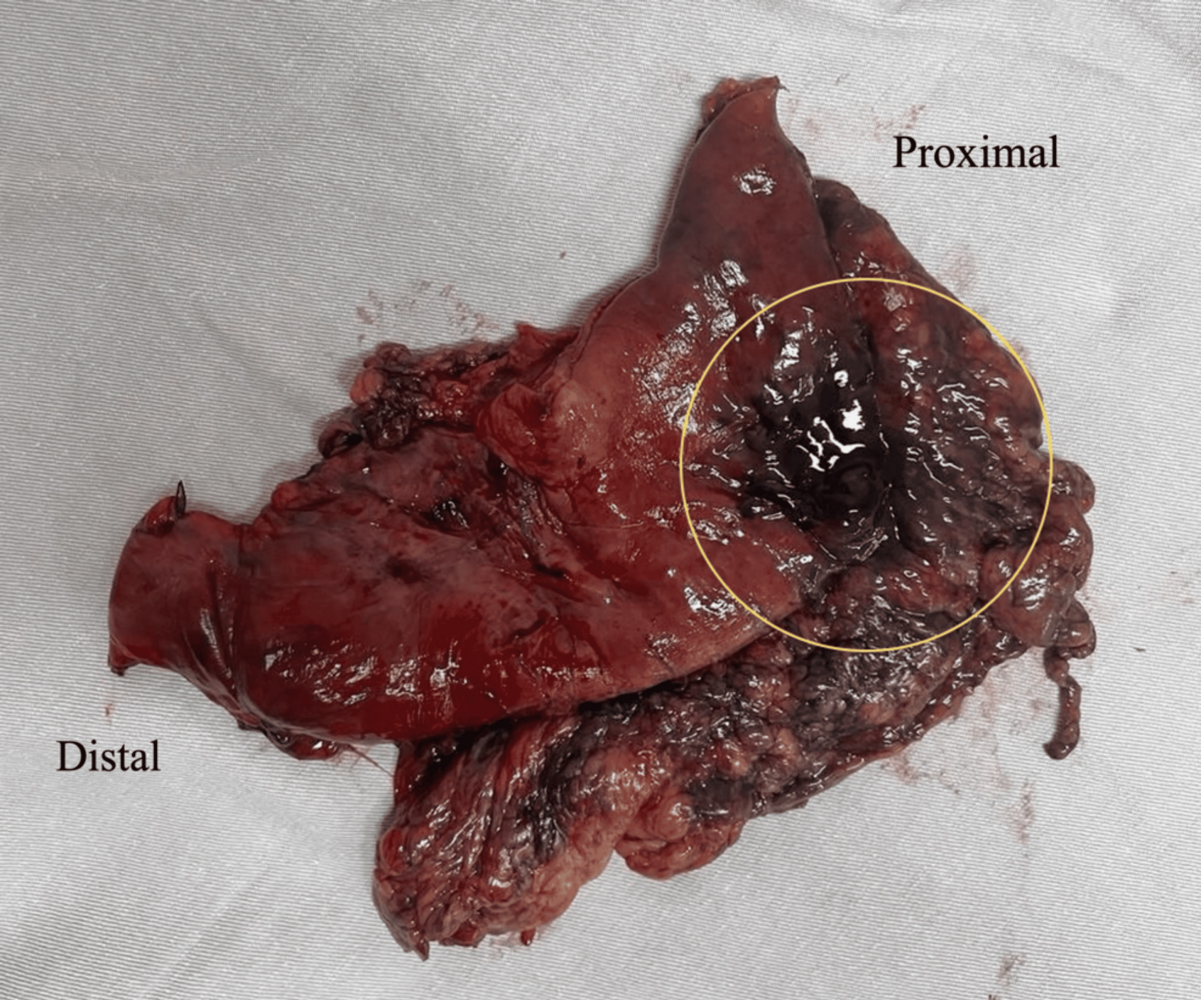
Image of the surgical specimen - subtotal gastrectomy - showing the stomach with perforation and necrotic portion (yellow ring)

An emergent exploratory laparotomy was indicated, revealing a hiatal defect of approximately 6 cm, with the stomach herniated into the thorax, signs of strangulation, ischemia of the greater curvature, and a gastric perforation of about 3 cm. Subtotal gastrectomy was performed using a linear stapler, with stapling of the proximal and distal ends. The proximal stapling was performed at the level of the cardia (Figure 5). Diaphragmatic repair was done with non-absorbable suture, and a peritoneostomy was performed due to hemodynamic instability. 

After clinical stabilization, the second surgical procedure was performed three days after the initial surgery. During the second procedure, the abdominal cavity was reassessed, a Roux-en-Y gastroenteroanastomosis was carried out, and the abdominal wall was closed. A post-anastomosis nasoenteral tube was placed for early enteral nutrition; no separate feeding jejunostomy was used, and the patient was not maintained on total parenteral nutrition. The intercostal drain, placed after the first surgery, was removed five days postoperatively, following low output and radiographic evidence of lung re-expansion. Enteral nutrition was initiated on the day following the second surgical procedure. The patient remained in the intensive care unit for 19 days and had a total postoperative hospital stay of 26 days, being discharged in good clinical condition with outpatient follow-up.

## Discussion

The surgical correction of hiatal hernias with fundoplication is a widely performed and generally successful procedure. However, it is associated with postoperative complications, some of which may manifest late. The most common complications include hernia recurrence, dysphagia, esophagitis, and persistent chest pain [11]. Recurrence of hiatal hernia can occur in up to 20% of patients and is often associated with inadequately corrected defects during the initial surgery or increased intra-abdominal pressure [12].

Early identification of these complications is crucial, as delays may lead to clinical deterioration, such as in the case of large or strangulated hernias, which may progress to intestinal loop suffering, ischemia, and perforation [13].

Strangulated hiatal hernia evolving with intrathoracic gastric perforation is a rare condition with the potential for high morbidity and mortality. Its clinical presentation is often nonspecific, mimicking thoracic pathologies such as pneumonia and pleural effusion, which can delay diagnosis and, consequently, definitive treatment [8]. In this case, the exteriorization of enteric content through thoracic drainage was a determining finding for therapeutic guidance.

Definitive treatment is usually surgical and aims to repair the hiatal hernia, correct the gastric perforation, and prevent future complications. The approach may vary depending on the location of the perforation, the presence of associated complications, and the patient’s clinical condition [9].

The prognosis depends on various factors, including the patient's age, comorbidities, time to diagnosis and treatment, and the presence of complications such as sepsis or multiple organ failure. The mortality rate can be significant, especially in patients with multiple comorbidities [14].

In hemodynamically unstable patients, as described in this case, a damage control surgical approach may be indicated. Peritoneostomy, one of the options within this strategy, allows for temporary control of the abdominal cavity until the patient is more stabilized for subsequent definitive intervention. The stepped approach strategy, combined with intensive clinical support, was crucial for the patient’s favorable evolution [15].

The case described illustrates the need for a high level of diagnostic suspicion in patients with a history of hiatal or diaphragmatic surgery who present with atypical chest symptoms. Early identification and prompt intervention are crucial to improving clinical outcomes and reducing the morbidity and mortality associated with this condition.

## Conclusions

This case exemplifies how late complications from surgical procedures can manifest in an atypical manner, requiring a careful approach and a high index of diagnostic suspicion to ensure effective management. In this patient, the early identification of an intrathoracic gastric perforation led to prompt surgical intervention with subtotal gastrectomy, followed by delayed reconstruction with a Roux-en-Y gastroenteroanastomosis after clinical stabilization. The postoperative management, including intensive care support and gradual reintroduction of enteral nutrition, contributed to a favorable outcome. The relevance of this case for emergency physicians and general surgeons is evident, as it highlights the importance of including this condition in the initial differential diagnosis, especially in patients with a history of abdominal and thoracic surgeries, where symptoms may not be clear. This scenario reinforces the need for a thorough analysis of the clinical presentation and the patient’s history to avoid severe complications and ensure a rapid and effective therapeutic response.
